# Supplementary material for: Exercise based Intervention For Metabolic Inflexibility Linked With Lipid Storage Myopathy Using Innovative CRISPR Etf-QO Mutant Knock-in Models
Source: bioRxiv. 2026 May 20:2026.05.18.726022. Preprint. [Version 1] doi: 10.64898/2026.05.18.726022 (PMC13228621; doi:10.64898/2026.05.18.726022)
Supplement: Supplement 1 [file NIHPP2026.05.18.726022v1-supplement-1.pdf]

bioRxiv preprint doi: https://doi.org/10.64898/2026.05.18.726022; this version posted May 20, 2026. The copyright holder for this preprint (which was not certified by peer review) is the author/funder, who has granted bioRxiv a license to display the preprint in perpetuity. It is made available under a CC-BY 4.0 International license.

Supplementary Information

Exercise based Intervention For Metabolic Inflexibility Linked Lipid Storage Myopathy Using Innovative CRISPR *Etf-QO* Mutant Knock-in Models

Sachin Budhathoki[a], Yiming Guo[a], Mary Doamekpor[a], and Girish Melkani[a,b,*]

[a]Department of Pathology, Division of Molecular and Cellular Pathology, Heersink School of Medicine, Heersink School of Medicine, The University of Alabama at Birmingham, AL 35294, USA. [b]UAB Nathan Shock Center, The University of Alabama at Birmingham, AL 35294, USA

*Correspondence Department of Pathology, Division of Molecular and Cellular Pathology, School of Medicine, University of Alabama at Birmingham, AL 35294, USA. Tel.: 1-205-996-0591; Fax: 1-205-934-7447; E-mail: girishmelkani@uabmc.edu

[#]Corresponding Author: E-mail: girishmelkani@uabmc.edu

Number of figures: 5

bioRxiv preprint doi: https://doi.org/10.64898/2026.05.18.726022; this version posted May 20, 2026. The copyright holder for this preprint (which was not certified by peer review) is the author/funder, who has granted bioRxiv a license to display the preprint in perpetuity. It is made available under a CC-BY 4.0 International license.

Fig. SI1

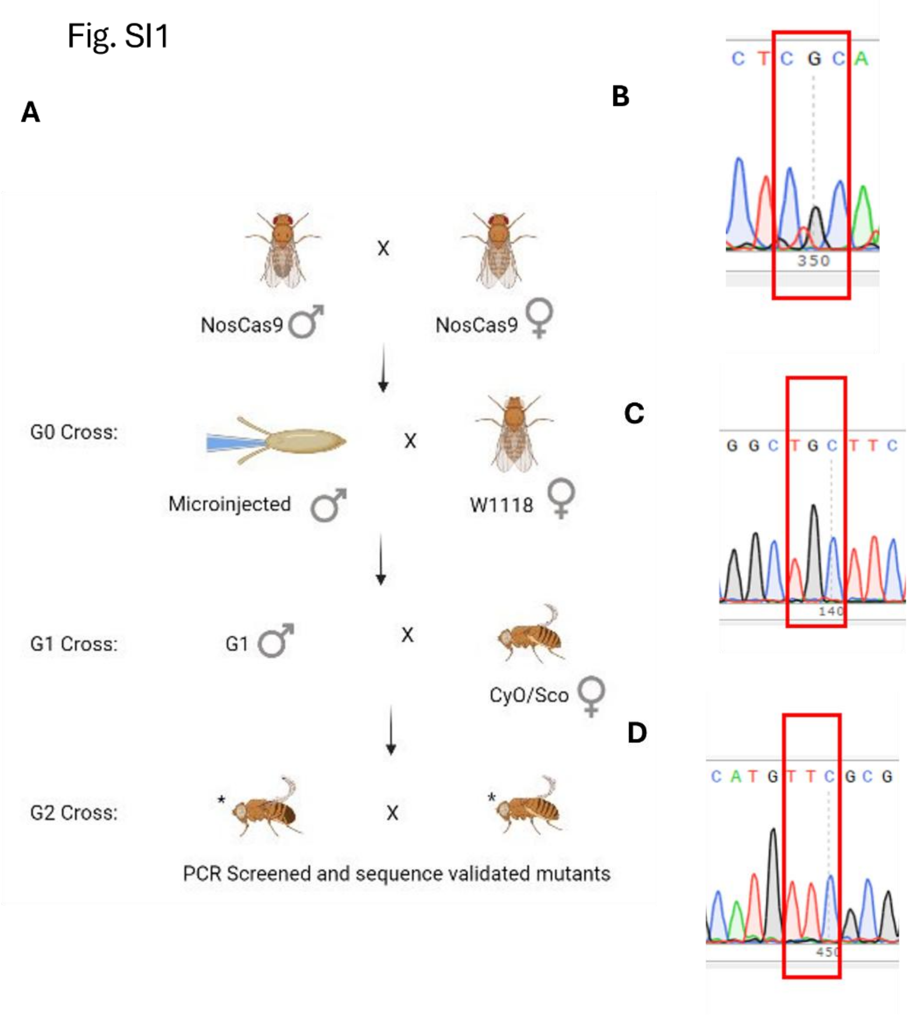

Figure SI1. Generation and validation of CRISPR knock-in Drosophila models carrying ETFQO point mutations associated with Multiple Acyl-CoA Dehydrogenase Deficiency (MADD). **(A)** Schematic representation of the CRISPR/Cas9-based workflow for introducing targeted point mutations in the *Etf-QO* gene. sgRNA-target plasmids and donor templates containing the desired mutations were microinjected into nos-Cas9 embryos (G0), followed by successive genetic crosses using balancer chromosomes to establish stable mutant lines. **(B–D)** Representative Sanger sequencing chromatograms confirming the presence of specific point mutations: L127R **(B)**, S296C **(C)**, and L399F **(D)**, located within conserved FAD and ubiquinone (UQ) binding domains of ETFQO.

bioRxiv preprint doi: https://doi.org/10.64898/2026.05.18.726022; this version posted May 20, 2026. The copyright holder for this preprint (which was not certified by peer review) is the author/funder, who has granted bioRxiv a license to display the preprint in perpetuity. It is made available under a CC-BY 4.0 International license.

Fig. SI2

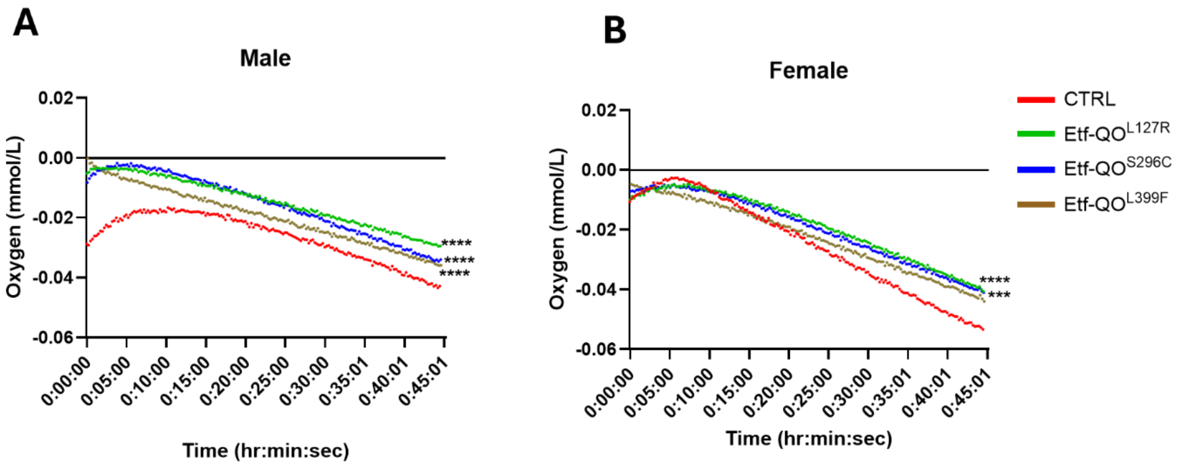

Figure SI2. Assessment of the effect of ETF-QO mutantion in mitochondrial respiration. Oxygen concentration profiles over time in exercise-conditioned male (A) and female (B) flies from control (CTRL) and ETF-QO mutant genotypes (**L127R**, **S296C**, **L399F**) measured using the MicroResp™ respirometry system (Loligo Systems). Each curve represents mean oxygen concentration (mmol/L) recorded at 1-second intervals for 45 minutes under standardized conditions. Mutants exhibited lower oxygen consumption rates compared to controls, indicating impaired respiratory capacity. Blank chambers served as negative controls. Data was analyzed using MicroResp software. Sample data corrected by subtracting the blank mean oxygen value from individual oxygen value across time. n = 5 flies per group. One-way ANOVA with Dunnett's multiple comparisons test, **p<0.01, ***p<0.001, ***p<0.0001

bioRxiv preprint doi: https://doi.org/10.64898/2026.05.18.726022; this version posted May 20, 2026. The copyright holder for this preprint (which was not certified by peer review) is the author/funder, who has granted bioRxiv a license to display the preprint in perpetuity. It is made available under a CC-BY 4.0 International license.

Fig. SI3

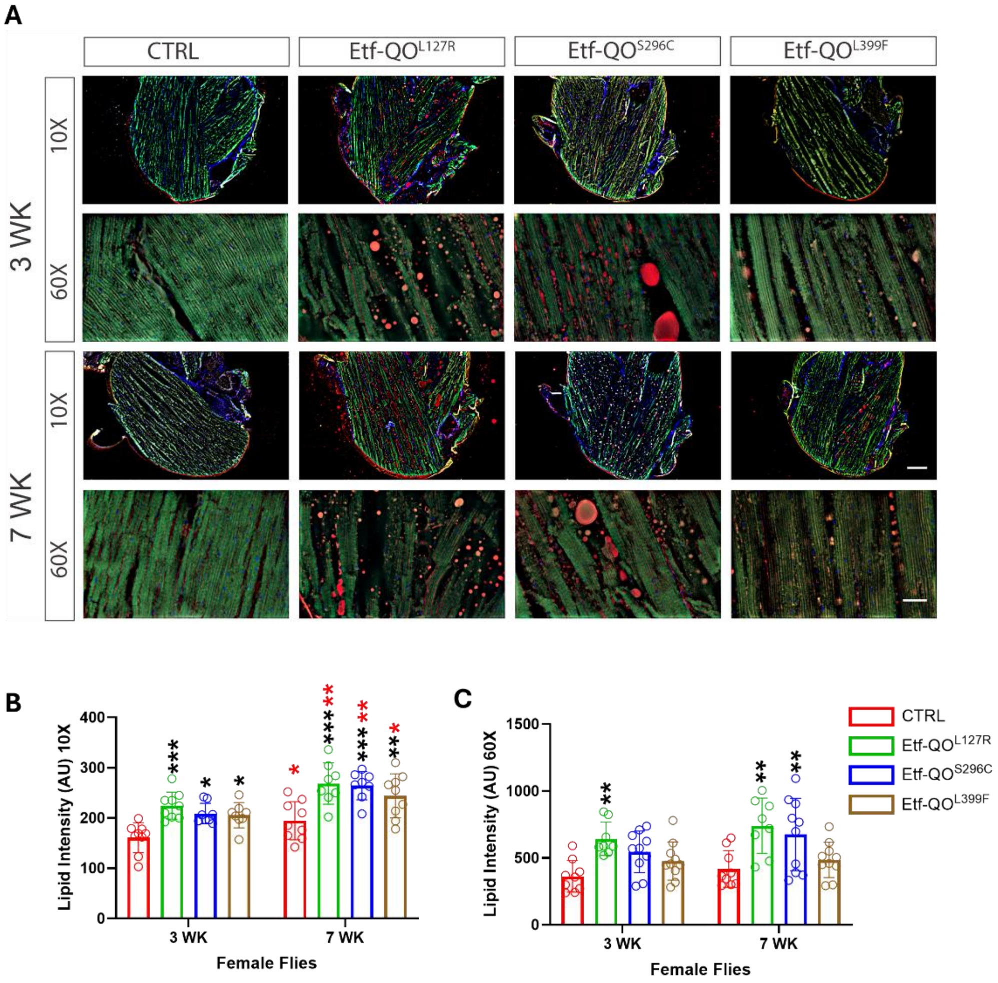

Figure SI3. Representative sagittal cryosections of thoracic muscles from control and Etf-QO mutant female flies at mid age (3 weeks) and old age (7 weeks) age, imaged at 10X and 60X magnification **(A)**. Sections were stained with phalloidin (green) to visualize actin cytoskeleton and DAPI (blue) for nuclei. Lipid droplets appear as red puncta. Mutants exhibit markedly higher lipid accumulation compared to controls, with severity slightly increasing at 7 weeks **(B)**. Quantification of lipid intensity at 10X **(B)** and 60X **(C)** magnification confirms significant elevation in lipid deposition in mutants relative to controls across both ages. (n=10–20 images, 5 females/group). Two-way ANOVA with Tukey's multiple comparisons test, $^{**}p<0.01$, $^{***}p<0.001$. Scale bar:100 μm(10X), 20 μm(60X).

bioRxiv preprint doi: https://doi.org/10.64898/2026.05.18.726022; this version posted May 20, 2026. The copyright holder for this preprint (which was not certified by peer review) is the author/funder, who has granted bioRxiv a license to display the preprint in perpetuity. It is made available under a CC-BY 4.0 International license.

Fig. SI4

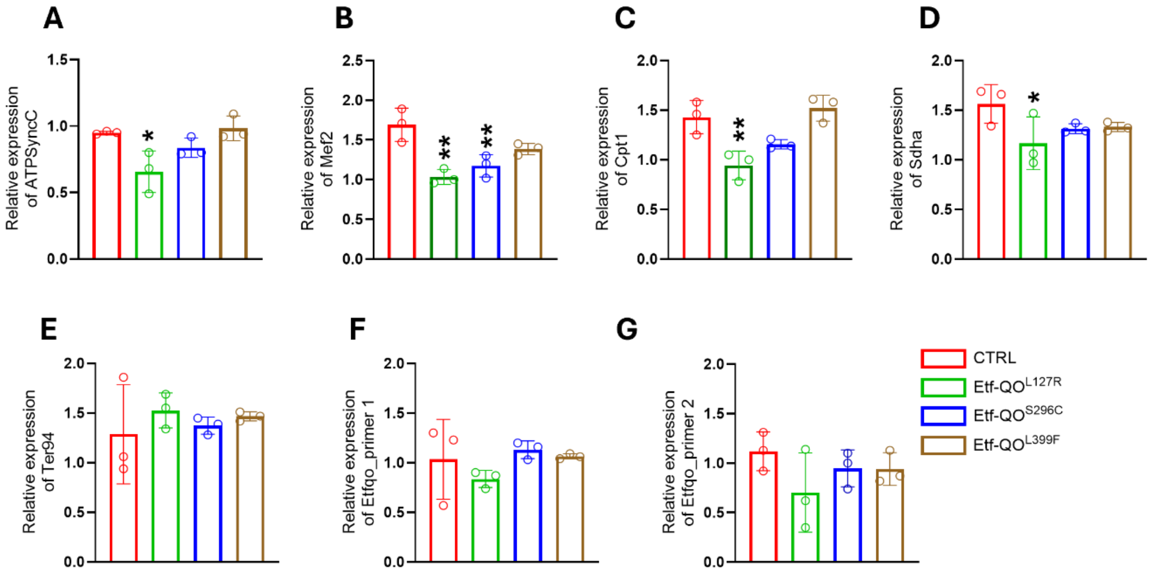

Figure SI4. Relative mRNA expression from RT-PCR data. Expression level of ATPsynC was comparable across lines (**A**). MEF2 expression was reduced in L127R and S296C compared with control (**B**). CPT1 and Sdha showed a downward expression in L127R (**C, D**). Ter94 expression did not show a consistent difference from control (**E**) and expression of EtfQO was comparable across all genotypes using two independent primer sets, indicating that the knock-in do not markedly alter steady-state EtfQO transcript levels (**F, G**). Expression normalized to Rpl11; data represent mean ± SD from three biological and two technical replicates obtained from thorax samples of 3-week-old males. One-way ANOVA with Dunnett's multiple comparisons test, *p<0.05, **p<0.01

bioRxiv preprint doi: https://doi.org/10.64898/2026.05.18.726022; this version posted May 20, 2026. The copyright holder for this preprint (which was not certified by peer review) is the author/funder, who has granted bioRxiv a license to display the preprint in perpetuity. It is made available under a CC-BY 4.0 International license.

Fig. SI5

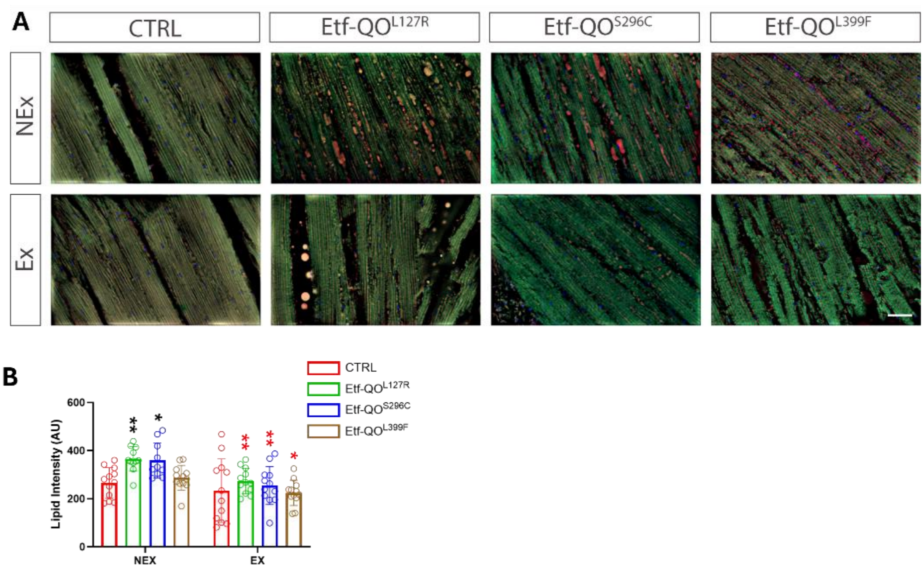

Figure SI5. **Effect of moderate exercise on lipid accumulation in indirect flight muscles of ETF-QO mutants**. Representative fluorescent images of thoracic indirect flight muscles from control (CTRL) and ETF-QO mutant flies under non-exercise (NEX) and exercise (EX) conditions **(A)**. Muscles were stained with phalloidin (green) to visualize actin filaments; lipid droplets appear as red puncta. Regular exercise (15 min/day for 2.5 weeks) visibly reduced lipid deposition in all mutant genotypes compared to sedentary cohorts. Scale bar: 100 μm. Quantification of lipid object count **(B)** and lipid object area **(C)** from IFM images. (n=10–20 images, 5 males/group). Two-way ANOVA with Tukey's multiple comparisons test, **p<0.01, ***p<0.001. Black and red asterisks denote comparison across genotypes and exercise conditions, respectively. Scale bar: 20 μm
